# Interferon-α2b Treatment for COVID-19

**DOI:** 10.3389/fimmu.2020.01061

**Published:** 2020-05-15

**Authors:** Qiong Zhou, Virginia Chen, Casey P. Shannon, Xiao-Shan Wei, Xuan Xiang, Xu Wang, Zi-Hao Wang, Scott J. Tebbutt, Tobias R. Kollmann, Eleanor N. Fish

**Affiliations:** ^1^Department of Respiratory and Critical Care Medicine, Union Hospital, Tongji Medical College, Huazhong University of Science and Technology, Wuhan, China; ^2^Prevention of Organ Failure (PROOF) Centre of Excellence and Centre for Heart Lung Innovation, St Paul's Hospital, University of British Columbia, Vancouver, BC, Canada; ^3^Division of Respiratory Medicine, Department of Medicine, University of British Columbia, Vancouver, BC, Canada; ^4^Systems Vaccinology, Center for Precision Health, Telethon Kids Institute, Nedlands, WA, Australia; ^5^Toronto General Hospital Research Institute, University Health Network and Department of Immunology, University of Toronto, Toronto, ON, Canada

**Keywords:** interferon, COVID-19, viral shedding, IL-6, inflammation, ARDS

## Abstract

The global pandemic of COVID-19 cases caused by infection with SARS-CoV-2 is ongoing, with no approved antiviral intervention. We describe here the effects of treatment with interferon (IFN)-α2b in a cohort of confirmed COVID-19 cases in Wuhan, China. In this uncontrolled, exploratory study, 77 adults hospitalized with confirmed COVID-19 were treated with either nebulized IFN-α2b (5 mU b.i.d.), arbidol (200 mg t.i.d.) or a combination of IFN-α2b plus arbidol. Serial SARS-CoV-2 testing along with hematological measurements, including cell counts, blood biochemistry and serum cytokine levels, and temperature and blood oxygen saturation levels, were recorded for each patient during their hospital stay. Treatment with IFN-α2b with or without arbidol significantly reduced the duration of detectable virus in the upper respiratory tract and in parallel reduced duration of elevated blood levels for the inflammatory markers IL-6 and CRP. These findings suggest that IFN-α2b should be further investigated as a therapy in COVID-19 cases.

## Introduction

In December 2019, an outbreak of pneumonia was reported in Wuhan, Hubei province, China, resulting from infection with a novel coronavirus (CoV), severe acute respiratory syndrome (SARS)-CoV-2. SARS-CoV-2 is a novel, enveloped betacoronavirus with phylogenetic similarity to SARS-CoV (1). Unlike the coronaviruses HCoV-229E, HCoV-OC43, HCoV-NL63, and HCoV-HKU, that are pathogenic in humans and are associated with mild clinical symptoms, SARS-CoV-2 resembles both SARS-CoV and Middle East respiratory syndrome (MERS), with the potential to cause more severe disease. A critical distinction is that CoVs that infect the upper respiratory tract tend to cause a mild disease, whereas CoVs that infect both upper and lower respiratory tracts (such as SARS-CoV-2 appears to be) may cause more severe disease. Coronavirus disease (COVID)-19, the disease caused by SARS-CoV-2, has since spread around the globe as a pandemic.

In the absence of a SARS-CoV-2-specific vaccine or an approved antiviral, a number of antivirals are currently being evaluated for their therapeutic effectiveness. Type I IFNs-α/β are broad spectrum antivirals, exhibiting both direct inhibitory effects on viral replication and supporting an immune response to clear virus infection (2). During the 2003 SARS-CoV outbreak in Toronto, Canada, treatment of hospitalized SARS patients with an IFN-α, resulted in accelerated resolution of lung abnormalities (3). Arbidol (ARB) (Umifenovir) (ethyl-6-bromo-4-[(dimethylamino)methyl]-5-hydroxy-1-methyl-2 [(phenylthio)methyl]-indole-3-carboxylate hydrochloride monohydrate), a broad spectrum direct-acting antiviral, induces IFN production and phagocyte activation. ARB displays antiviral activity against respiratory viruses, including coronaviruses (4).

Herein we report on the clinical course of disease in 77 confirmed cases of COVID-19 admitted to Union Hospital, Tongii Medical College, Wuhan, China, treated with interferon (IFN)-α2b, ARB, or a combination of IFN-α2b plus ARB.

## Materials and Methods

### Patients and Treatments

Individuals with suspected COVID-19 were admitted to Union Hospital, Tongii Medical College, Wuhan, China, during the period January 16–February 20, 2020, based on initial symptoms that included fever, chills, cough, sore throat, headache, nasal discharge, myalgia, fatigue, shortness of breath and/or diarrhea. Each patient was asked to identify their date of symptom onset. At the discretion of the attending physician, laboratory confirmed COVID-19 cases received antiviral treatment with either IFN-α2b (Tianjin Sinbobioway Biology, 5 mIU/ml), ARB (arbidol hydrochloride; Jiangsu Simcere Pharm. Co., 100 mg dispersible tablets), or a combination of IFN-α2b plus ARB, in accordance with the current practice guidelines at the hospital at that time. 5 mIU IFN-α2b (1 ml) were added to 2 ml of sterile water and introduced as an aerosol by use of a nebulizer and mask. IFN-α2b treatment was bid, i.e., 10 mIU/day. ARB treatment was 200 mg (2 tablets) tid, i.e., 600 mg/day. Additional COVID-19 confirmed cases from Wuhan Temporary Shelter Hospital (February 2–17, 2020), who were transferred to Union Hospital and treated with only ARB, were also included in this study. Ethics approval for analysis of all data collected was waived by hospital Institutional Review Boards, since all patient data collected conformed with the policies for a public health outbreak investigation of emerging infectious diseases issued by the National Health Commission of the People's Republic of China.

### Laboratory Tests

Throat swab specimens were tested by real time polymerase chain reaction (RT-PCR) for SARS-CoV-2. The SARS-CoV-2 laboratory test assay employed was based on the Centers for Diseases Control & Prevention, U.S.A. (CDC) recommendation (5). Briefly, throat-swab specimens from the upper respiratory tract of patients suspected of having SARS-CoV-2 infection were placed into collection tubes prefilled with 150 μL of virus preservation solution and total RNA was extracted using a respiratory sample RNA isolation kit (High Pure Viral RNA Kit. Roche, Basel, Switzerland). RT-PCR assays for SARS-CoV-2 RNA were conducted using two target genes, namely open reading frame1ab (ORF1ab) and nucleocapsid protein (N). Samples were designated positive (+) or negative (-) based on a threshold adjusted to fall within the PCR exponential phase, for both target genes. Complete blood count and serum biochemical tests were assessed as per the Union Hospital's routine clinical laboratory procedures. Serum cytokine levels (IL-2, IL-4, IL-6, IL-10, TNF-α, IFN-γ) were assayed using the BD Biosciences Th1/Th2 cytokine kit, as per the manufacturer's instructions (BD Ltd., Franklin Lakes, NJ, USA) and peripheral blood cell populations enumerated using a BD FACSCanto Plus flow cytometer as per the Union Hospital's routine clinical laboratory protocols.

### Statistical Analysis

All analyses were carried out using R version 3.6.0 (6). Descriptive statistics (Table 1 and group means reported in the text) and figures convey the data as-is, but all time-to-event and time course analyses were adjusted for age, sex, and the presence of one or more comorbidities. Note that age was coded as either a continuous variable or binary variable (with > 50 or > 60 as the threshold), and results are reported across all three variations.

**Table 1:** Demographics and clinical characteristics of patient cohort.

	**IFN *n* = 7**	**IFN+ARB *n* = 46**	**ARB *n* = 24**	***P*-value**
Age, years	41.3 (27–68)	40.4 (25–80)	64.5 (37–73)	<0.001
Male (%)	0 (0.0%)	20 (43.5%)	11 (45.8%)	0.076
Female (%)	7 (100%)	26 (56.5%)	13 (54.2%)	
Co-morbidities (%)^a^	14.3%	15.2%	54.2%	0.002
**Initial symptoms**				
Fever (%)	57.1%	58.7%	70.8%	0.632
Cough (%)	42.9%	50.0%	54.2%	0.888
Fatigue (%)	14.3%	23.9%	37.5%	0.422
Myalgia (%)	14.3%	13.0%	29.2%	0.228
Headache (%)	14.3%	6.52%	4.17%	0.590
Pharyngalgia (%)	0.00%	13.0%	8.33%	0.742
Chest pain (%)	14.3%	6.52%	20.8%	0.134
Expectoration (%)	14.3%	8.70%	20.8%	0.281
Nausea (%)	0.00%	0.00%	4.17%	0.403
Diarrhea (%)	14.3%	4.35%	20.8%	0.081
Days from symptom onset to hospital admission^b^	8.0 [5.5, 15.5]	6.5 [3.0, 10.0]	10.0 [4.5, 19.5]	0.087
Days from symptom onset to 1^st^ treatment^b^	8.0 [6.5, 16.0]	8.0 [5.25, 11.0]	17.0 [10.0, 22.0]	<0.001

a *Hypertension, diabetes, COPD, chronic bronchitis, heart disease, cancer*.

b *Median and interquartile range [Q1, Q3] is reported*.

### Time-to-Event Analysis

Time-to-viral clearance, defined as the number of days elapsed from the onset of symptoms to the time of the first of two consecutive negative PCR tests at least 24 h apart, was compared among the treatment groups using time-to-event analysis. Date of onset of symptoms was considered as date of onset of disease, an appropriate time point to allow for interrogation of disease course for all patients in this COVID-19 cohort. The statistical significance of treatment was assessed using Cox proportional hazards.

### Time Course Analysis

Time course data were aligned to date of symptom onset and aggregated over 2–4-day intervals (depending on the analyte) to account for data not being available for all patients at all time points during disease course. If time course plots diverged between treatment groups, to test whether these observations met statistical significance, analysis of variance (ANOVA) was used to test for treatment effect.

## Results

### Clinical and Laboratory Data: Moderate COVID-19 Disease

Table 1 summarizes the patient demographics and clinical characteristics of the cohort of COVID-19 cases evaluated in this exploratory study. 77 adults with confirmed COVID-19 admitted to Union Hospital, Wuhan, and at the discretion of the admitting physician, were treated with nebulized IFN-α2b (*n* = 7), ARB (*n* = 24) or a combination treatment of IFN-α2b plus ARB (*n* = 46); IFN-α2b and ARB treatments were standard of care practice at this time at Union Hospital, Wuhan. For 50% of all cases, treatment was started within 72 h of confirmation of infection by a PCR(+) result; for 75% of cases, treatment started within 96 h of a PCR(+) test and for 95% of cases, within 10 days of PCR(+). While all patients received various prophylactic antibiotics, there was no case of proven or suspected bacterial infection.

Serial clinical evaluations were performed on all patients. Irrespective of the treatment group, none of the patients evaluated in this study exhibited persistent signs or symptoms of end organ dysfunction. Specifically, none of the patients developed respiratory distress requiring prolonged oxygen supplementation or intubation; consequently, none of the patients in this cohort required intensive care. Outside of the admission temperature, when ~50% of all patients exhibited fever (temperature > 38°C; which was successfully treated with ibuprofen), no other occurrence of fever was noted irrespective of antiviral treatment group (Supplementary Figure 1). While all patients showed some radiographic abnormalities on chest computer tomography (CT) that were interpreted by local radiologists as “consistent with viral pneumonia,” detailed evaluation of the CT findings were not performed due to the overwhelming workload at Union Hospital at the time of this study. Serial laboratory measurements of blood levels for hemoglobin, glucose, total bilirubin, direct bilirubin, alanine aminotransferase (ALT), aspartate aminotransferase (AST), lactate dehydrogenase (LDH), creatine kinase (CK), blood urea nitrogen (BUN), albumin (Alb), creatinine, and troponin I were also conducted (Supplementary Figure 2). Beyond a mild transaminitis (ALT elevation) early during hospitalization, which subsequently improved in all patients, the data for blood chemistries indicated that levels fluctuated closely around the limits of normal over the course of hospitalization, without a clear or consistent difference among treatment groups. Peripheral blood cell populations, including total white blood cells (WBC), lymphocyte, CD4+ T cell, CD8+ T cell, B lymphocyte, neutrophil, NK cell and platelet counts were also measured over the course of hospitalization (Supplementary Figure 3). With the exception of elevated platelets, which peaked two weeks into the disease course, all other cell populations fluctuated around the normal range with no clear or consistent difference discernible among antiviral treatment groups. Together, the clinical and laboratory data indicate that the entire cohort evaluated in this study consisted of moderate cases of COVID-19 across all treatment groups.

Clinical course of the COVID-19 cases was also assessed in relation to age, sex and co-morbidities. With the exception of hemoglobin, which was lower in females, for each of the other measurements listed above, age, sex and co-morbidity differences in the treatment groups did not shift values out of normal range.

### Effects of IFN Treatment on Viral Clearance

**Figure 1:**
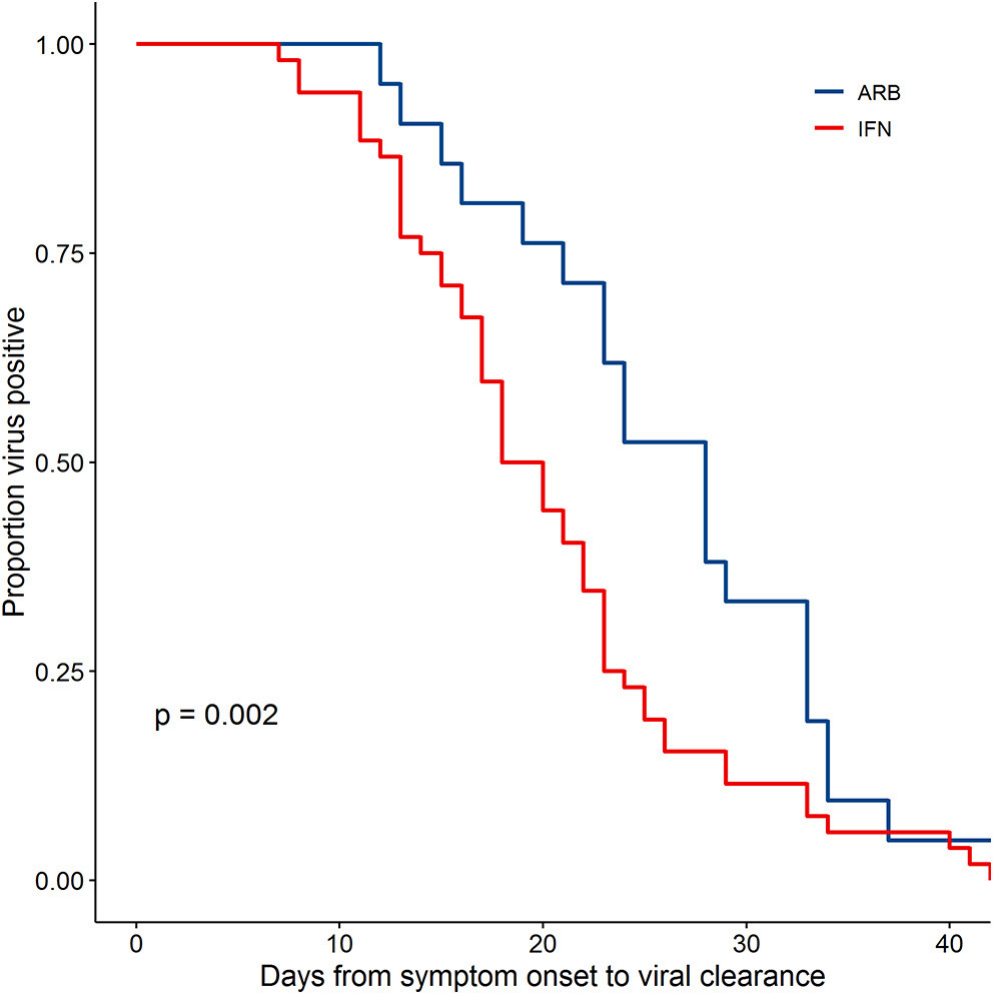
IFN-α2b treatment accelerated viral clearance. Confirmed COVID-19 cases were treated either with ARB alone (ARB; 24 patients) or IFN-α2b with or without ARB (IFN; 53 patients). Upper respiratory samples were assessed by PCR for the presence of SARS-CoV-2. Shown is the proportion of patients that had detectable virus as a function of the day of sampling from symptom onset. Empirical survival curves are shown here, while the *p*-value for treatment effect was assessed using a Cox proportional-hazards model that included age and co-morbidities as covariates.

Viral clearance was defined as two consecutive negative PCR tests at least 24 h apart as previously described (5). Assessing disease course from Day of symptom onset (D0) to the first negative (-) PCR of 2 consecutive PCR (-)s, the data revealed a significantly different rate of viral clearance for each treatment group (Supplementary Figure 4). Specifically, outcome analysis suggested that treatment with IFN-α2b, whether alone or in combination with ARB, accelerated viral clearance when compared to ARB treatment alone. Mean days to viral clearance were 27.9 for ARB alone treated patients, 21.1 days for those treated with IFN alone and 20.3 days for those treated with IFN + ARB (from onset of symptoms). Closer scrutiny of the treatment regimens for those cases treated with a combination of IFN-α2b and ARB revealed that for 16 of the 46 cases (34.8%) IFN-α2b treatment was started after ARB treatment had been initiated and, for 24 cases (52.2%), IFN-α2b treatment was continued after ARB treatment was stopped (Supplementary Figure 5). Given the heterogeneity of treatment regimens within this treatment group, we considered the time to viral clearance for all cases treated with IFN (i.e., combined the IFN-only with the IFN plus ARB group) compared to those who received ARB only. The data shown in Figure 1 reveal the statistically significant accelerated viral clearance from the upper respiratory tract in patients who received IFN-α2b treatment (20.4 days, *p* = 0.002). i.e., IFN treatment accelerated viral clearance by ~7 days.

### Effects of IFN Treatment on Circulating Cytokine Levels and Biomarkers of Inflammation

**Figure 2:**
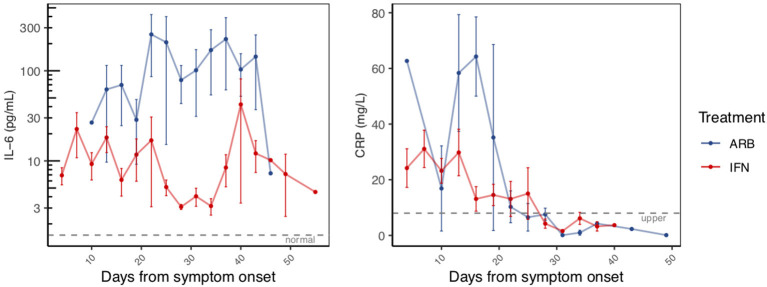
Reduced inflammatory markers with IFN-α2b treatment. The same patients as in Figure 1 were serially sampled for assessment of interleukin-6 (IL-6; LHS panel) and C-reactive protein (CRP; RHS panel) from the day of symptom onset. Values recorded were aggregated across 3 day intervals and shown as the mean +/– S.E.

Circulating cytokine levels (IL-2, IL-4, IL-10, IFN-γ, IL-6, TNFα) and biomarkers of inflammation (C-reactive protein, CRP and procalcitonin, PCT) were also examined over the disease course. Circulating levels of PCT, IL-2, IL-4, IL-10, IFN-γ, and TNFα remained within their normal range throughout disease course, irrespective of treatment group (Supplementary Figure 6). Notable and significant exceptions were IL-6 and CRP. As disease course progressed and prior to resolution, we observed a clear distinction of serum IL-6 levels between cases treated with IFN (i.e., IFN alone or IFN + ARB) and cases treated with ARB alone. More specifically, whereas circulating levels of IL-6 remained low for all patients who received IFN, those who received ARB alone (i.e., with no IFN) exhibited a significant spike in circulating IL-6 levels (Figure 2, Supplementary Figure 7). Specifically, over the time period day 12 to day 42 (from onset of symptoms), on average patients in the ARB only group had higher IL-6 levels than the patients treated with IFN alone or a combination of IFN + ARB, by 33.5 pg/mL.

As for treatment effects on viral clearance, whether the effects of treatment were analyzed from date of onset of symptoms or date of treatment onset, IFN treatment significantly reduced circulating IL-6 levels. We also noted elevated levels of CRP in the cohort (Supplementary Figure 7). Similar to IL-6, CRP also returned to within normal range as disease resolved. Our data suggest that treatment with IFN, whether alone or in combination with ARB, reduced the circulating CRP levels (Figure 2). Specifically, over the time period day 0–20 (from onset of symptoms) on average patients in the ARB only group had higher CRP levels than the patients treated with IFN alone or a combination of IFN + ARB, by 25.7 mg/L.

### Effects of Age, Co-morbidities and Sex on Treatment Outcomes

Co-morbidities did not significantly affect the effects of IFN treatment on time to viral clearance (*p* = 0.371), or IL-6 (*p* = 0.456), and CRP (*p* = 0.420) levels. Cognizant that the ARB-only treatment group consisted generally of older patients, we adjusted for age in the statistical analyses. Age was significant as a covariate for CRP (*p*-values ranged 1.2 × 10^−5^ to 4.5 × 10^−6^) and sometimes for IL-6 (*p*-values ranged 0.02–0.07). Regardless of whether age was considered as a continuous variable or a categorical variable (<50 yrs vs. >50 yrs; <60 yrs vs. >60 yrs), the effects of IFN treatment on IL-6, CRP, and time to viral clearance all remained statistically significant. For those cases treated with ARB alone, IL-6 levels were significantly higher than for those treated with IFN from day 12–42 (*p*-values ranged from 1.1 × 10^−9^ to 7.7 x 10^−10^ depending on age coding). Similarly, for the ARB alone treatment group, CRP levels were significantly higher than for those cases treated with IFN from day 0–20 (*p*-values ranged 0.0032–0.0037 depending on age coding). Time to viral clearance was significantly shorter for those cases treated with IFN-α2b (alone and in combination with ARB) compared to those treated only with ARB (*p* = 0.0018) after adjustment for age and co-morbidities. With adjustment for age and co-morbidities, the effects of IFN-α2b treatment (alone and in combination with ARB) remained significant for reducing circulating levels of IL-6 (*p* = 7.7 × 10^−10^) and CRP (*p* = 0.0035).

The contributions of sex to the differences in outcomes observed could not be comprehensively evaluated, since information on pre- vs. post- menopause, phase of menstrual cycle, or contraceptive use, variables that independently may influence immune responses to COVID-19, was not collected. Nevertheless, when sex was only considered in the context of male vs. female, although sex influenced treatment outcomes, these effects did not negate or eliminate the statistical significance of the effects of IFN treatment on viral clearance and IL-6 and CRP levels. Sex was significant as a covariate for viral clearance (*p* = 0.026) and for CRP (*p* = 0.0001), but not for IL-6 (*p* = 0.084). With adjustment for age, co-morbidities and sex, the effects of IFN-α2b treatment (alone and in combination with ARB) remained significant for accelerated viral clearance (*p* = 0.0019), and reducing circulating levels of IL-6 (*p* = 5.7 × 10^−10^) and CRP (*p* = 0.0022).

## Discussion

This uncontrolled, exploratory study provides several important and novel insights into COVID-19 disease. Importantly, IFN-α2b therapy appears to shorten duration of viral shedding. Reduction of markers of acute inflammation such as CRP and IL-6 correlated with this shortened viral shedding, suggesting IFN-α2b acted along a functional cause-effect chain where virally induced inflammation represents a pathophysiological driver. Taken together, these findings support the plausibility of IFN-α2b representing a therapy for COVID-19 disease.

As the SARS-CoV-2 pandemic takes an ever-increasing toll, the urgent search for effective prophylactic and therapeutic interventions is rapidly accelerating. This includes lopinavir/ritonavir (7, 8), chloroquine (9), remdesivir (10), as well as IFN-α/β (2) and ARB (4) and combinations of these. Most of these antivirals only have *in vitro* data to support consideration for coronavirus targets prior to clinical testing; as such, while unfortunate, it is not surprising that there is a high chance of failure (11). However, we had shown during the SARS-CoV-1 outbreak in Canada that IFN-α treatment could hasten resolution of coronavirus-mediated human disease (3). This prompted us to evaluate IFN-α therapy for COVID-19 disease in the early stages of the outbreak in Wuhan, Hubei province, China. Indeed, our analysis suggests that inhaled IFN-α2b accelerated viral clearance from the respiratory tract and hastened resolution of systemic inflammatory processes when compared to ARB treatment alone. Notably, a recent publication reported that ARB treatment, at the same doses used in this study, did not affect the rate of viral clearance in non-ICU patients hospitalized with COVID-19 compared with untreated patients (12). While we recognize that our data are at best suggestive, given the urgency, the findings indicate that a follow-up randomized clinical trial (RCT) is now warranted. Success may not only benefit the individual infected patient but, by reducing duration of viral shedding even in moderate cases (such as this cohort), assist in slowing the population spread.

The reduction of the inflammatory biomarker IL-6 following inhaled IFN-α2b therapy not only supported a clinically relevant impact of this approach, but also hinted at likely functional connections between viral infection and host end organ damage. IL-6 has been shown to provide prognostic value in acute respiratory distress syndrome (ARDS), which is the most severe form of COVID-19 disease (13). If this were indeed the case, then targeting interventions such as interleukin-6 (IL-6) receptor inhibitors (e.g., tocilizumab or sarilumab) toward this axis may prove a useful therapeutic adjunct, at least in those most severely ill. This form of therapy has recently been approved by China's National Health Commission^1^ and is currently under consideration by the Italian Medical Agency^2^. The advantage of IFN-α2b over blocking IL-6 rests in IFN targeting the cause (SARS-CoV-2), not only the symptoms (IL-6).

This exploratory study has several significant limitations. Most obvious is the fact that the study cohort was small, non-randomized, with unbalanced demographics between treatment arms that were of unequal size. There were disparities in age, sex and co-morbidities between the IFN treated and ARB treated cases. However, the effects of IFN treatment on accelerated viral clearance and reductions in circulating IL-6 and CRP levels remained significant after adjusting for age, sex and co-morbidities. Notably, we considered this an exploratory study only, with the objective of determining in as rapid a manner as possible if a full trial should be considered. The results indicate that an IFN-α RCT is now warranted. Furthermore, since the entire cohort consisted only of moderate cases of COVID-19 disease, our findings may not be indicative of what occurs in more severely ill patients; such caution about generalizability is indeed further supported by the limited impact of age, sex and comorbidities on the course of COVID-19 disease in our cohort, as each of these have been shown to have varying degrees of influence on clinical course (14).

Irrespective of these significant limitations, to our knowledge, the findings presented here are the first to suggest therapeutic efficacy in COVID-19 disease of IFN-α2b, an available antiviral intervention. Furthermore, beyond clinical benefit to the individual patient, treatment with IFN-α2b may also benefit public health measures aimed at slowing the tide of this pandemic, in that duration of viral shedding appears shortened.

## Data Availability Statement

The raw data supporting the conclusions of this article will be made available by the authors, without undue reservation, to any qualified researcher.

## Ethics Statement

Ethical review and approval was not required for the study on human participants in accordance with the local legislation and institutional requirements. Written informed consent for participation was not required for this study in accordance with the national legislation and the institutional requirements.

## Author Contributions

QZ was responsible for patient care and treatment, clinical oversight, and clinical data collection. VC and CS analyzed the data and generated the figures. X-SW, XX, XW, and Z-HW collected laboratory and radiographic data. ST analyzed data. TK and EF conducted data analysis, data interpretation, literature searches, and manuscript writing.

### Conflict of Interest

The authors declare that the research was conducted in the absence of any commercial or financial relationships that could be construed as a potential conflict of interest.

## Footnotes

^1^https://www.reuters.com/article/us-health-coronavirus-china-roche-hldg/china-approves-use-of-roche-arthritis-drug-for-coronavirus-patients-idUSKBN20R0LF

^2^https://112.international/society/italy-claims-to-find-drugs-against-coronavirus-49605.html
