# Corrigendum: Interferon-α2b Treatment for COVID-19

**DOI:** 10.3389/fimmu.2020.615275

**Published:** 2020-10-27

**Authors:** Qiong Zhou, Virginia Chen, Casey P. Shannon, Xiao-Shan Wei, Xuan Xiang, Xu Wang, Zi-Hao Wang, Scott J. Tebbutt, Tobias R. Kollmann, Eleanor N. Fish

**Affiliations:** ^1^ Department of Respiratory and Critical Care Medicine, Union Hospital, Tongji Medical College, Huazhong University of Science and Technology, Wuhan, China; ^2^ Prevention of Organ Failure (PROOF) Centre of Excellence & Centre for Heart Lung Innovation, St Paul’s Hospital, University of British Columbia, Vancouver, BC, Canada; ^3^ Division of Respiratory Medicine, Department of Medicine, University of British Columbia, Vancouver, BC, Canada; ^4^ Systems Vaccinology, Center for Precision Health, Telethon Kids Institute, Nedlands, WA, Australia; ^5^ Toronto General Hospital Research Institute, University Health Network & Department of Immunology, University of Toronto, Toronto, ON, Canada

**Keywords:** interferon, COVID-19, viral shedding, IL-6, inflammation, ARDS

## Error in Figure/Table

In the original article, there was a mistake in **Table 1** as published. For ‘Days from symptom onset to treatment,’ the IFN+ARB and ARB values were inadvertently switched. IFN+ARB should be 8.0 [5.0,11.0] and ARB should be 17.0 [10.0, 22.0]. The corrected **Table 1** appears below.

The authors apologize for this error and state that this does not change the scientific conclusions of the article in any way. The original article has been updated.

**Table 1:** Demographics and clinical characteristics of patient cohort.

	IFN n = 7	IFN+ARB n = 46	ARBn = 24	p-value
Age, yrs	41.3 (27–68)	40.4 (25–80)	64.5 (37–73)	<0.001
Male (%) Female (%)	0 (0.0%) 7 (100%)	20 (43.5%) 26 (56.5%)	11 (45.8%) 13 (54.2%)	0.076
Comorbidities (%)^a^	14.3%	15.2%	54.2%	0.002
**Initial symptoms**				
Fever (%)	57.1%	58.7%	70.8%	0.632
Cough (%)	42.9%	50.0%	54.2%	0.888
Fatigue (%)	14.3%	23.9%	37.5%	0.422
Myalgia (%)	14.3%	13.0%	29.2%	0.228
Headache (%)	14.3%	6.52%	4.17%	0.590
Pharyngalgia (%)	0.00%	13.0%	8.33%	0.742
Chest pain (%)	14.3%	6.52%	20.8%	0.134
Expectoration (%)	14.3%	8.70%	20.8%	0.281
Nausea (%)	0.00%	0.00%	4.17%	0.403
Diarrhea (%)	14.3%	4.35%	20.8%	0.081
Days from symptom onset to hospital admission^b^	8.0 [5.5, 15.5]	6.5 [3.0, 10.0]	10.0 [4.5, 19.5]	0.087
Days from symptom onset to 1^st^ treatment^b^	8.0 [6.5, 16.00]	8.0 [5.25, 11.0]	17.0 [10.0, 22.0]	<0.001

a Hypertension, diabetes, COPD, chronic bronchitis, heart disease, cancer.

b Median and interquartile range [Q1, Q3] are reported.

