# Supplementary material for: Interferon-α2b Treatment for COVID-19
Source: Front Immunol. 2020 May 15;11:1061. doi: 10.3389/fimmu.2020.01061 (PMC7242746; doi:10.3389/fimmu.2020.01061)
Supplement: Supplementary file 1 [file Data_Sheet_1.PDF]

SUPPLEMENTARY DATA

Interferon-α2b treatment for COVID-19

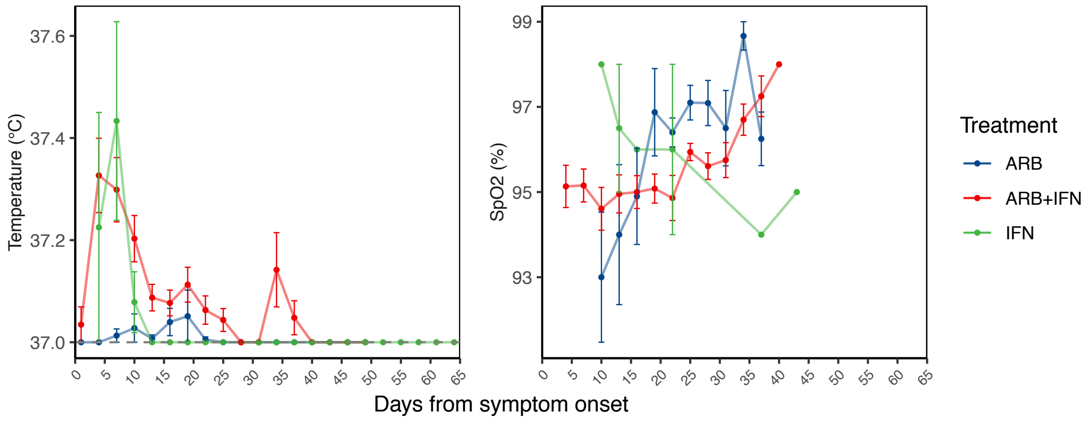

Figure 1: No differences in vital signs according to treatment group.

Temperature and peripheral capillary oxygen saturation (SiPO2) over the course of COVID-19 disease according to treatment group. Values recorded were aggregated across the day intervals indicated and are shown as mean+/- SE.

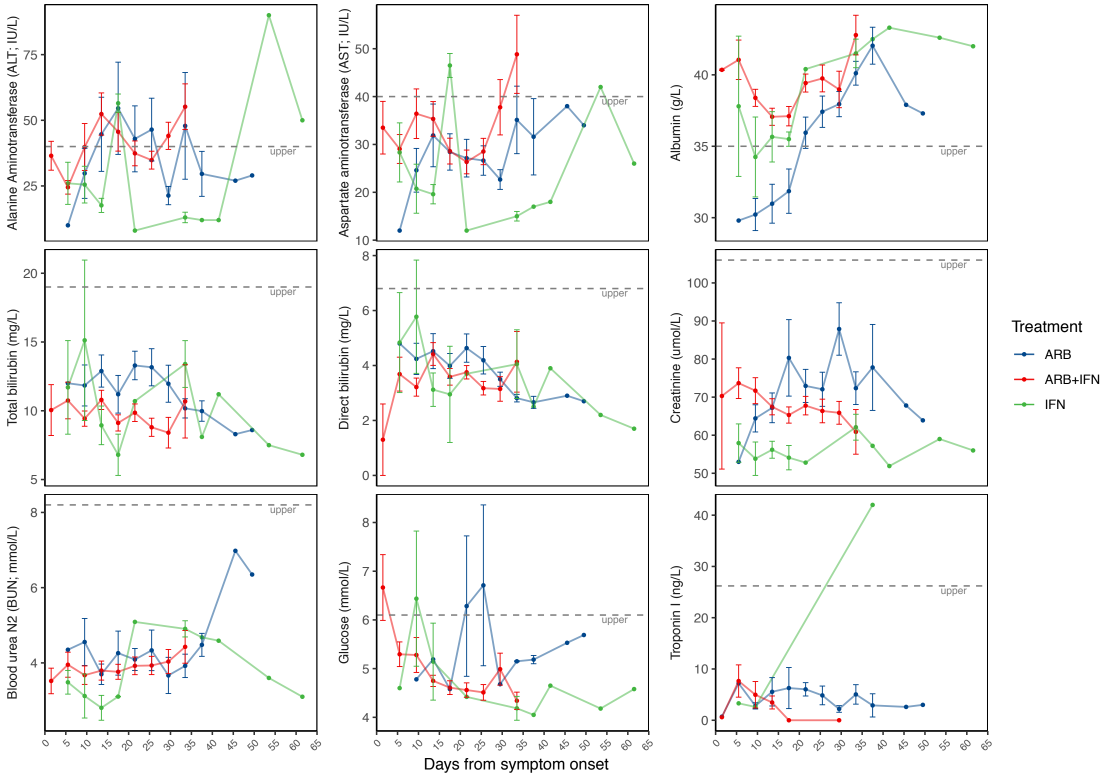

Figure 2: **No differences in blood chemistries according to treatment group.** Blood chemistries of haemoglobin, glucose, total bilirubin, direct bilirubin, alanine aminotransferase (ALT), aspartate aminotransferase (AST), lactate dehydrogenase (LDH), creatine kinas (CK), blood urea nitrogen (BUN), albumin (Alb), creatinine, and troponin 1 over the course of COVID-19 disease according to treatment group. Values recorded were aggregated across the day intervals indicated and are shown as mean+/- SE. Upper limits of normal range in healthy adults are indicated as dashed lines.

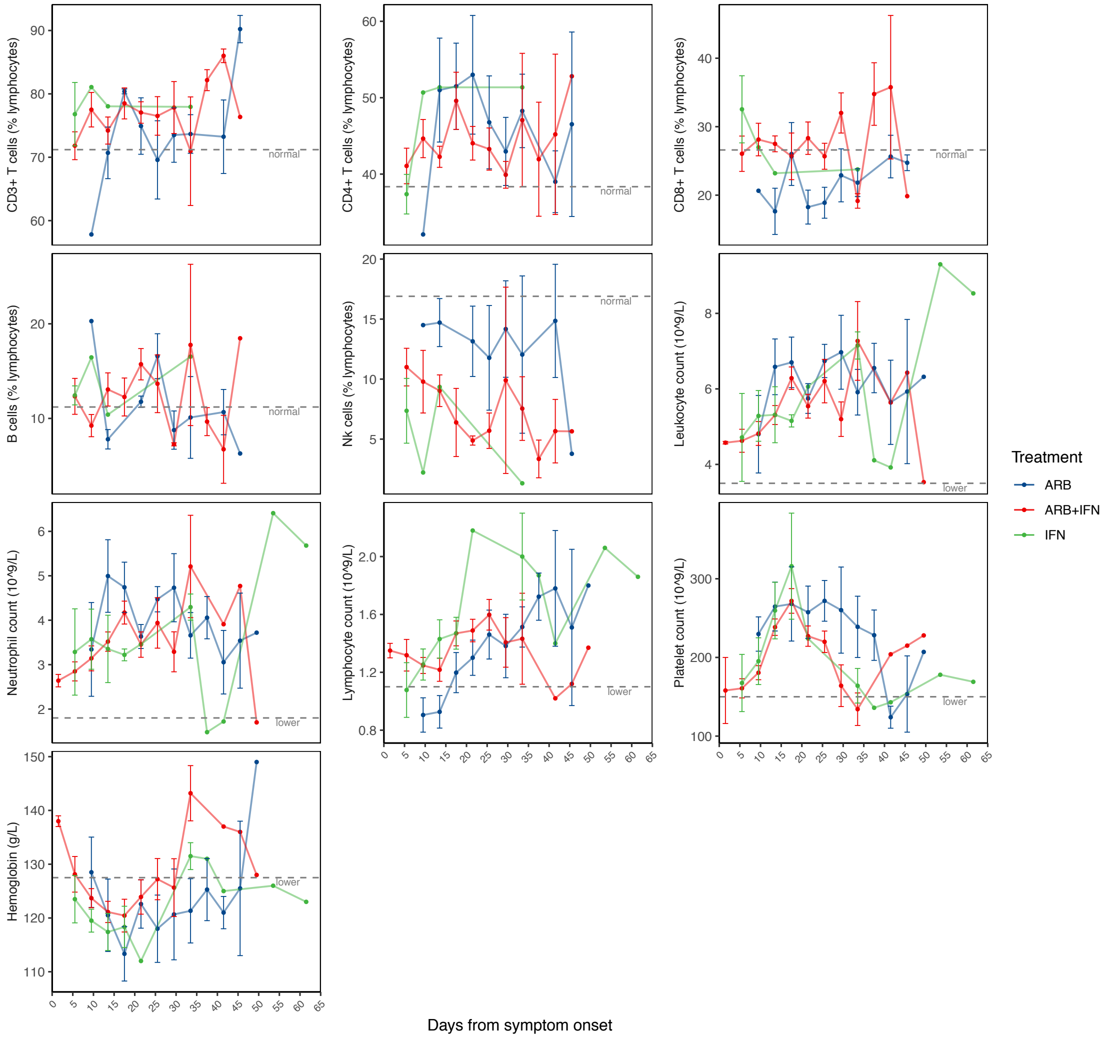

Figure 3: **No differences in peripheral blood cell populations according to treatment group.** Peripheral blood cell populations, including total white blood cells (WBC), lymphocyte, CD4+ T cell, CD8+ T cell, B lymphocyte, neutrophil, NK cell and platelet counts over the course of COVID-19 disease according to treatment group. Values recorded were aggregated across the day intervals indicated and are shown as mean+/- SE. For the dashed lines, normal indicates the mean of the normal range, lower indicates the lower limit of the normal range, in healthy adults.

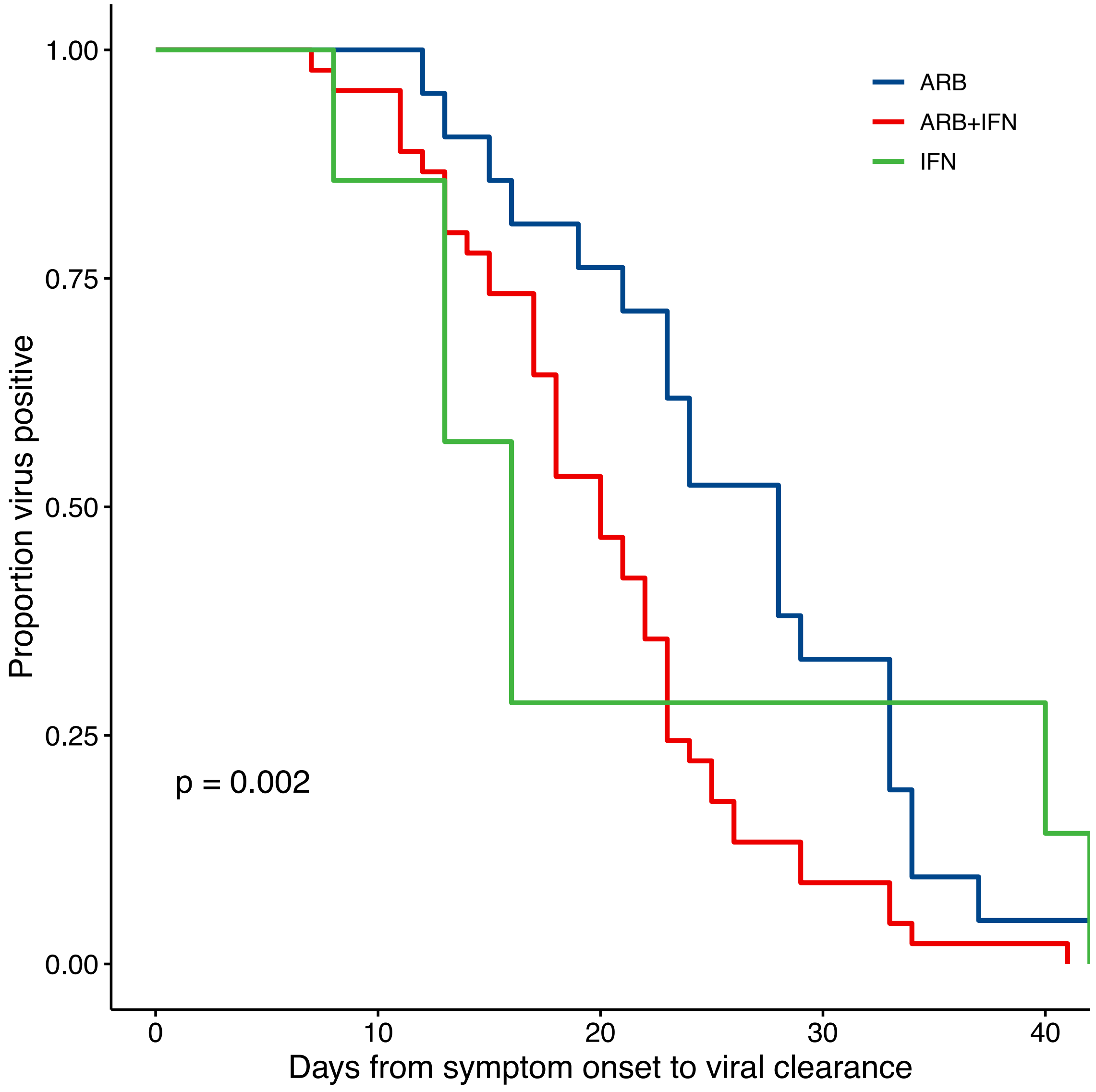

Figure 4: **IFN-α2b treatment accelerated viral clearance**

Confirmed COVID-19 cases were treated either with ARB alone (ARB; 24 patients) or IFN-α2b alone (IFN; 7 patients) or ARB with IFN-α2b (ARB+IFN; 46 patients). Upper respiratory samples were assessed by PCR for the presence of SARS-CoV-2. Shown is the proportion of patients that had detectable virus as a function of the day of sampling from symptom onset.

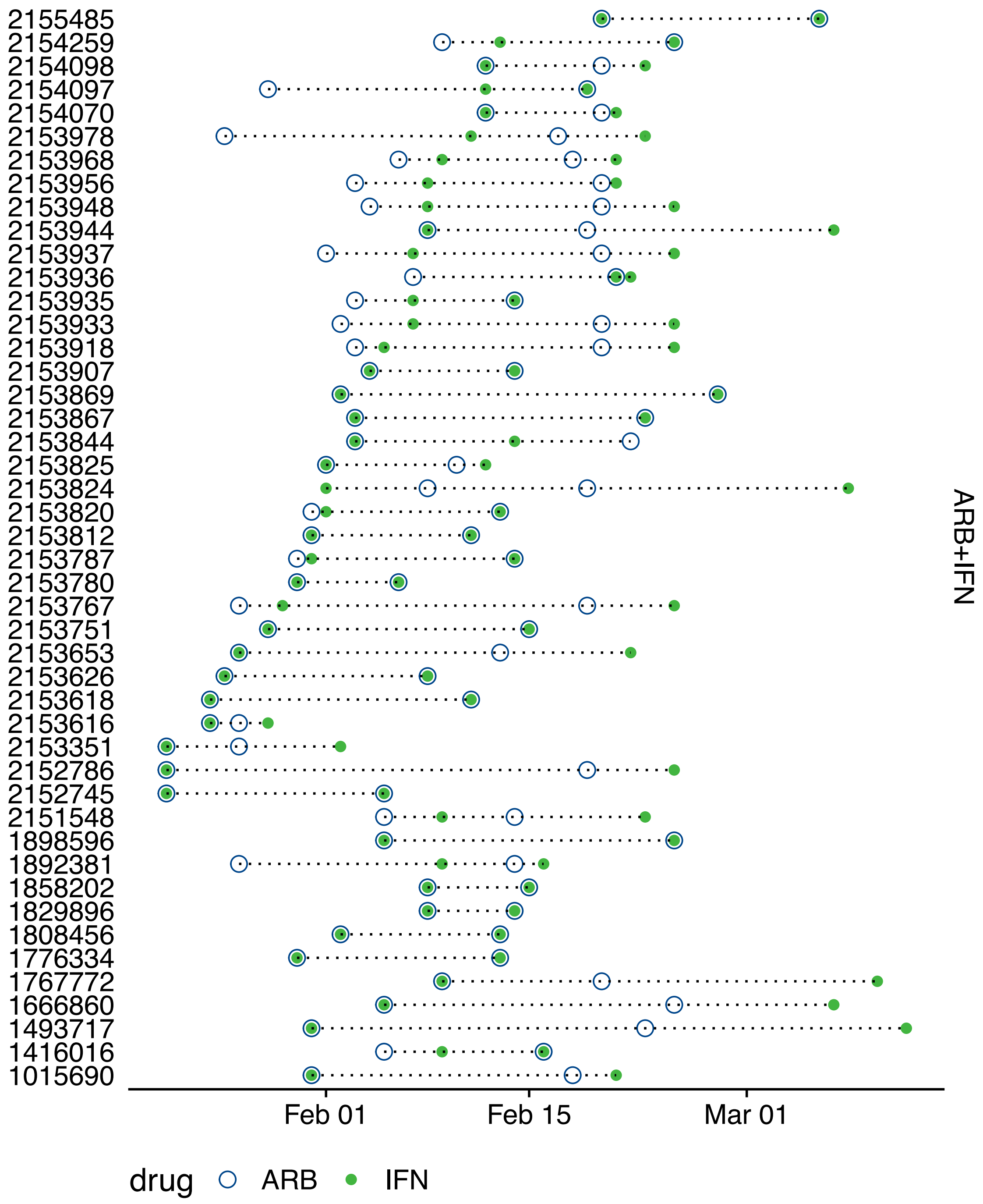

Figure 5: **Treatment start and stop dates for patients treated with ARB plus IFN-α2b.** For each patient identified on the Y-axis (ID number), start and end dates of treatment with ARB (open circle) and IFN-α2b (green filled circle) are shown, with the total treatment time represented as the dotted line.

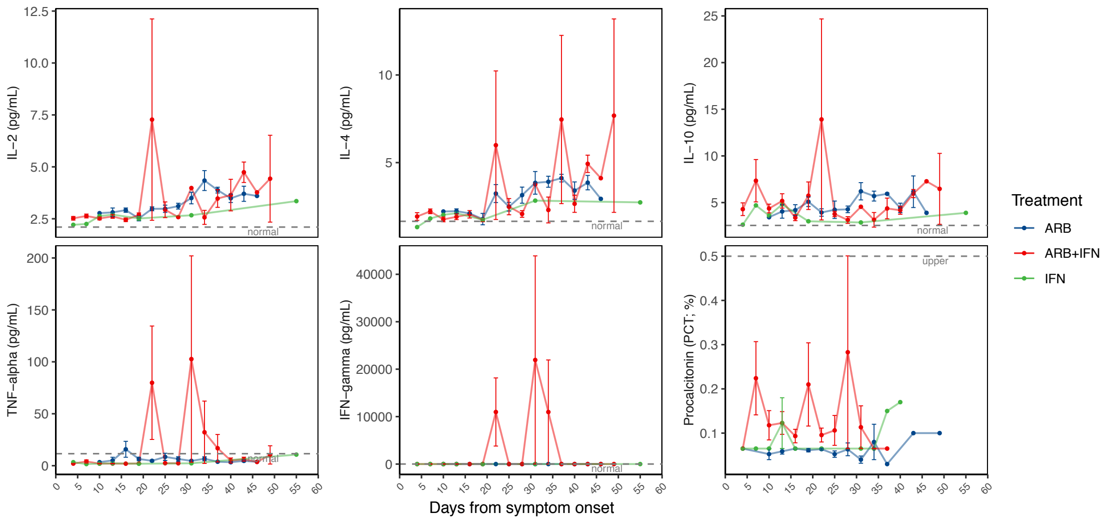

Figure 6: **No differences in blood cytokine levels and PCT according to treatment group.** Periheral blood levels of IL-2, IL-4, IL-10, IFN-γ TNF and PCT over the course of COVID-19 disease according to treatment group. Values recorded were aggregated across the day intervals indicated and are shown as mean+/- SE. Dashed lines indicate the normal levels in healthy adults and, for procalcitonin, the upper limit of normal range.

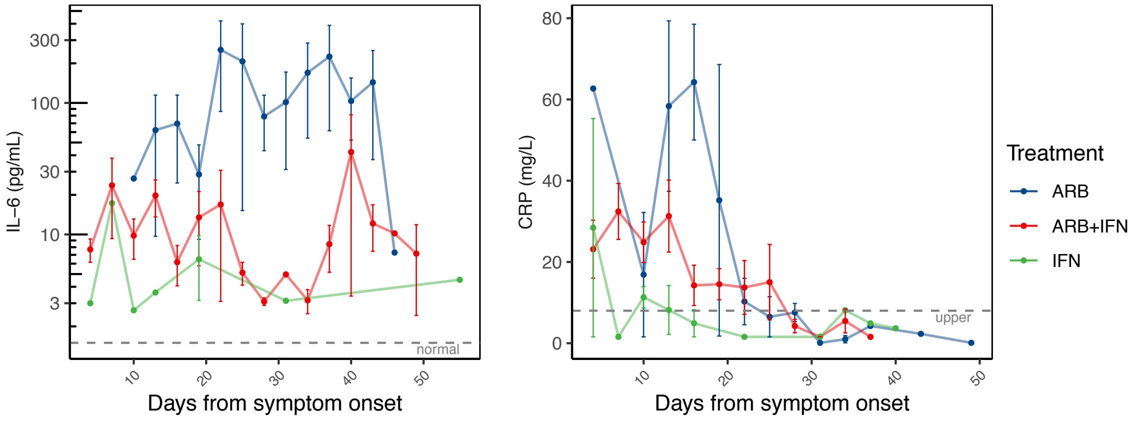

Figure 7: Reduced inflammatory markers with IFN-α2b treatment. Confirmed COVID-19 cases were treated either with ARB alone (ARB; 24 patients) or IFN-α2b (IFN; 7 patients) or ARB with IFN-α2b (ARB+IFN; 46 patients). Peripheral blood levels of IL-6 (A) and CRP (B) from the day of symptom onset. Values recorded were aggregated across the day intervals indicated and are shown as mean+/- SE. The dashed line for IL-6 indicates the normal level in healthy adults, and for CRP, the upper limit of the normal range.
